# Micro-Organisms of Sewage

**Published:** 1895-02-16

**Authors:** 


### MICRO-ORGANISMS OF SEWAGE.

DURING the last few years the Main Drainage Committee of the London County Council has been engaged in investigating the micro-organisms contained in the sewers. Hitherto the reports have been chiefly concerned with the micro-organisms contained in sewer gas; but the report which has just been issued deals entirely with the bacterial flora of the sewage itself. The report is too long to enter into minute detail, but one or two important facts have been added to our knowledge, and these we will summarise as shortly as possible.

It is highly improbable that such bacteria as are found in sewer gas have escaped from the fluid contents of the sewers, because the most common variety found in the sewage is seldom or never to be found in the surrounding air. The bacteria of sewer air and those of sewage present different proportions of micrococci and bacilli; and, while moulds exist in vast quantities in the air, they are rarely to be found in the fluid contents of the drains.

The collaborateurs of this important report make a most valuable suggestion with regard to a possible source of the ill-effects which have been ascribed to sewer air. They think it possible that the subsoil may become polluted by constant infiltration of excrementitious matter through leaky drains; parts of this may become dry and capable of giving off particulate germs to the currents of passing air, and of thus finding access to rooms of dwelling-houses.

Most exhaustive examinations have been made by Mr. Laws and Dr. Andrewes to determine whether the germs of typhoid fever can be detected in ordinary sewage. Their results are almost entirely negative. Bacillus typhosus was indeed discovered at the outfall of a fever hospital, where a considerable number of cases of enteric fever were quartered, but subsequent dilution in the main arteries of the sewers rendered the percentage of these pathogenic germs one that could hardly be mathematically stated, much less practically or experimentally determined. Moreover, from careful laboratory researches, Mr. Laws and Dr. Andrewes are able to state that the bacillus of enteric fever thrives very badly on a nutrient medium which consists either of sterilised sewage or sewage containing other forms of micro-organisms. The joint authors of this report conclude with the following sentence: "That sewage-polluted soils may give up germs to the subsoil air is possible, but that the air of sewers themselves should play any part in the conveyance of typhoid fever appears to us as the result of investigations in the highest degree unlikely."

Although this report makes no practical suggestions as to a means of checking epidemics of enteric fever, it is to be hoped that it will for ever put to sleep certain fallacies regarding the influence of sewer gases in the etiology of typhoid fever which on insufficient scientific data have found a firm footing in public belief.

